# Formation of a Mixed-Species Biofilm Is a Survival Strategy for Unculturable Lactic Acid Bacteria and *Saccharomyces cerevisiae* in *Daqu*, a Chinese Traditional Fermentation Starter

**DOI:** 10.3389/fmicb.2020.00138

**Published:** 2020-02-06

**Authors:** Yi Fan, Xiaoning Huang, Jingyu Chen, Beizhong Han

**Affiliations:** ^1^College of Food Science and Nutritional Engineering, China Agricultural University, Beijing, China; ^2^Key Laboratory of Viticulture and Enology, Ministry of Agriculture and Rural Affairs, Beijing, China

**Keywords:** mixed-species biofilm, *Saccharomyces cerevisiae*, *Lactobacillus*, alcoholic fermentation starter, unculturable

## Abstract

The existence and function of unculturable microorganisms are necessary to explain patterns of microbial diversity and investigate the assembly and succession of the complex microbial community. Chinese traditional alcoholic fermentation starter contains a complex microbial community harboring unculturable species that control the microbial diversity and have distinct functions. In this study, we revealed the presence, functions, and interactions of these unculturable species. Results of microbial diversity revealed by culture-dependent and metagenomic sequencing methods identified unculturable species and the potential functional species. Unculturable *Saccharomyces cerevisiae* and *Lactobacillus* sp. had a strong ability to form biofilms and co-existed as a mixed-species biofilm in the starter community. Using a hydrolase activity assay and fortified fermentation, we determined that the function of *S. cerevisiae* and *Lactobacillus* sp. to produce ethanol and flavor compounds. Widespread microbial interactions were identified among the biofilm isolates. *S. cerevisiae* was the main component of the biofilm and dominated the metabolic activities in the mixed-species biofilm. The environmental adaptability and biomass of *Lactobacillus* sp. were increased through its interaction with *S. cerevisiae*. The mixed biofilm of *S. cerevisiae* and *Lactobacillus* sp. also provides a tool for correlating microbial diversity patterns with their function in the alcoholic fermentation starter, and may provide a new understanding of fermentation mechanisms. Formation of a mixed-species biofilm represents a strategy for unculturable species to survive in competition with other microbes in a complex community.

## Introduction

The coexistence and interaction of multiple microorganisms in complex microbial communities is widespread in the environment (Smid and Lacroix, 2013; Wright et al., 2013; Banerjee and Schlaeppi, 2018). With the development of sequencing technology and bioinformatics analysis, amplicon sequencing and shotgun metagenomic sequencing have become powerful methods for revealing the abundance and functions of each species in the community (Faust and Raes, 2012; Mendes et al., 2015; Escobar-Zepeda et al., 2016). Due to the difficulties in isolating and cultivating species with low abundance, the culturable dominant species in communities became the main research focus. However, keystone taxa in the community can determine community composition and function irrespective of their abundance (Banerjee and Schlaeppi, 2018). Thus, keystone taxa could exist as unculturable, low-abundance species in a diverse community. For complex microbial communities, one of the biggest challenges is to isolate and cultivate the unculturable species, which is the foundation of community function and community reconstruction research.

Unculturable and low-abundance species commonly exist in many different environmental communities such as traditional alcoholic fermentation starter (TAFS) (Zheng et al., 2012; Bal et al., 2014; Browne et al., 2016). Chinese TAFS also known as *Daqu* is the saccharification and fermentation agent for *Baijiu* (Chinese liquor) (Zheng et al., 2011b; Jin et al., 2017). *Daqu* is made of barley, pea, and wheat through solid-state fermentation. The microbial community of *Daqu* has diverse species producing various enzymes to convert starch into alcohol and flavor compounds during alcoholic fermentation to make *Baijiu*. During alcoholic fermentation, *Saccharomyces cerevisiae* and lactic acid bacteria (LAB) are considered to be functional species due to their ability to produce ethanol and flavor compounds (Pérez-Martín et al., 2013; Michlmayr and Kneifel, 2014; Zhang et al., 2014; Filannino et al., 2018). However, based on results of culture-dependent and culture-independent methods, LAB were only detected in the starter using culture-independent methods and *S. cerevisiae* was not detected with all of the methods (Zheng et al., 2014; Zou et al., 2018). The prevalence of *S. cerevisiae* and unculturable LAB is uncertain in the starter community, which makes alcoholic fermentation starter a model community to explore the presence and functions of unculturable species. Because the fermentation mechanism of alcoholic fermentation starter is unknown, the manufacture of *Daqu* and *Baijiu* still depends on the experience of skilled workers, which presents a major challenge for industrial production.

Unculturable microorganisms tend to have a low prevalence or be slow-growing when co-existing with other species in the community, making them difficult to isolate (Pham and Kim, 2012; Steen et al., 2019), while their requirement for specific nutrients and incubation conditions makes them difficult to cultivate. However, unculturable microorganisms could be isolated with the development of technology. Common strategies include reducing microbial diversity before isolation and mimicking the original habitat of unculturable microorganisms (Pham and Kim, 2012). Numerous strategies have been implemented to reduce the microbial diversity of a complex community before isolation of unculturable species. These include density-gradient centrifugation methods and the design of selective media based on the rRNA sequence of target species (Oberhardt et al., 2015). The effects of those isolation and cultivation methods are highly dependent on the sample. In the case of alcoholic fermentation starter, the relevant limitations are the extremely low abundance of *S. cerevisiae* and the nutritional requirements of LAB. Because LAB and *S. cerevisiae* play dominant roles in alcoholic fermentation, some researchers have suggested these microorganisms may originate from sorghum (raw material for alcoholic fermentation) and the environment instead of the starter (Pang et al., 2018). However, an overlooked explanation for their existence is that they may survive as mixed-species biofilms.

A biofilm comprises a community of microbes associated with a surface, typically encased in an extracellular matrix (Monds and Toole, 2009). In natural environments, microorganisms tend to adhere to any solid surface immersed in a liquid medium and assemble in a complex multispecies consortium often embedded in extracellular polymeric substance (EPS) produced by the microorganisms known as mixed biofilm or multispecies biofilm (Hall-Stoodley et al., 2004; Wimpenny, 2009). Biofilm formation is one of the reasons for unculturable microorganisms (Vartoukian et al., 2010). With powerful tolerance for environmental stress and antibiotics, microorganisms can survive as a biofilm under adverse conditions (Giaouris et al., 2014; Feng et al., 2017). They can also exist as a biofilm during the food fermentation process, which has significant effects on the quality and flavor of fermented foods. Acetic acid bacteria form biofilm during strawberry vinegar fermentation to survive and produce acetic acid (Jesús et al., 2015). The biofilm formed by *Oenococcus oeni* during wine fermentation increases the tolerance to wine stress and improves functional performance with effective malolactic activities (Bastard et al., 2016). Previous studies proved that *S. cerevisiae* and *Lactobacillus plantarum* could form a mixed-species biofilm on a glass surface in liquid media (Nozaka et al., 2014; Furukawa et al., 2015). A mixed biofilm could therefore be the lifestyle for *S. cerevisiae* and LAB in TAFS community.

In this study, we used the alcoholic fermentation starter as a model microbial community to reveal the existence of unculturable keystone species. In particular, we report the presence, functions, and interactions of unculturable *S. cerevisiae* and *Lactobacillus* in TAFS. Analyses of microbial isolates and metagenomic sequencing data confirmed the uncultivability and potential functionality of *S. cerevisiae* and *Lactobacillus* sp. in starter community. We observed mixed biofilm formation and simulated the original environment to isolate and cultivate the above microorganisms. Biofilm formation assay, hydrolase activity analysis and fortified alcoholic fermentation were conducted to evaluate the function of unculturable species. Microbial interaction patterns in the co-culture of *S. cerevisiae* and *Lactobacillus* were investigated using the biofilm formation assay, spatial observation, and metabolite analysis. Our study demonstrates the formation of a mixed biofilm as a survival strategy for unculturable microorganisms, and highlights the underlying microbial interactions.

## Materials and Methods

### Shotgun Metagenomic Sequencing

The *Daqu* sample used in this study was the smashed ready-to-use *Daqu* for liquor fermentation, obtained from Shanxi Xinghuacun Fenjiu Distillery Co., Ltd. (Fenyang, Shanxi, China). After collection, the sample transferred to sterile bags, sealed, and stored at 4°C. Genomic DNA for library construction was extracted using an automated nucleic acid extractor (Bioteke Biotech Co., Ltd., Beijing, China).

Metagenomic DNA libraries were constructed using the Illumina TruSeq^TM^ DNA Sample Prep Kit, with an average insert size of 350 bp. The HiSeq 3000/4000 PE Cluster Kit and HiSeq 3000/4000 SBS Kits were used for paired-end sequencing (2 × 150 bp) with an Illumina Hiseq3000, at Majorbio Bio-Pharm Technology Co., Ltd. (Shanghai, China). The assembly of clean reads was performed using the SOAP *de novo* software (Version 1.3) for a range of k-mers (39∼47). The MetaGene program (Hideki et al., 2006) was used to predict the open reading frames (ORFs) within the contigs for the sequencing data. ORFs with a length greater than 100 bp were translated into amino acid sequences using the NCBI translation table. The abundance calculation was carried out using the SOAPaligner software (Version 2.20). The abundance of genes with greater than 95% identity was retained for statistical tests.

In this study, taxonomic annotation of species was performed using BLASTP (version 2.2.28+), which compared sequencing data to the NCBI nr database. The Kyoto Encyclopedia of Genes and Genomes (KEGG) database was used for functional annotation. For KEGG pathway annotation, BLAST (version 2.2.28+) was applied against the KEGG database with an *E*-value cutoff of 10^–5^, and the KOBAS 2.0 web server was used for functional annotation.

### Amplicon Sequencing and Isolation of Microorganisms

The V3–V4 region of the bacterial 16S ribosomal RNA gene was amplified using PCR with primers 338_F (3′-ACTCCTACGGG AGGCAGCA-5′) and 806_R (3′-GCACTACHVGGGTW TCTAAT-5′) (Quast et al., 2013). For fungi, the internal transcribed spacer (ITS2) regions were amplified with primers ZIT_F (3′-GCATCGATGAAGAACGCAGC-5′) and ZITS_R (3′-TCCTCCGCTTATTGATATGC-5′) (Toju et al., 2012). Amplicons were extracted from 2% agarose gels, purified using the AxyPrep DNA Gel Extraction Kit (Axygen Biosciences, Union City, CA, United States) according to the manufacturer’s instructions, and quantified using QuantiFluor^TM^ -ST (Promega, United States). Purified amplicons were pooled in equimolar amounts and paired-end sequenced (2 × 250) on an Illumina MiSeq platform following standard protocols. Raw fastq output files from the sequencer were demultiplexed and quality-filtered using QIIME (version 1.17). Operational taxonomic units (OTUs) were clustered with a 99% similarity cutoff using UPARSE (version 7.1) and chimeric sequences were identified and removed using UCHIME. Using the Ribosomal Database Project Classifier (version 2.2), the taxonomy of each 16S rRNA gene sequence was analyzed against the Silva 123/16S rRNA database with a 97% identity threshold. The fungal ITS sequence was clustered using USEARCH (version 7.1) and aligned by the BLAST algorithm with a 97% identity threshold.

To retrieve isolates from the mixed-species biofilm, 10 g *Daqu* powder was added to 90 mL *Daqu* medium consisting of 22.5 g/mL malt powder and 15.0 g/mL pea powder under facultatively anaerobic conditions for 72 h. *Daqu* medium was sterilized by autoclaving at a temperature of 121°C under 15 psi pressure for 15 min. Then LAB strains were isolated with MRSA and 0.1% w/v natamycin and incubated at 30°C for 72 h. Fungi were isolated with Yeast Peptone Dextrose medium (YPD), 1.5% of agar was added for solid medium and incubated at 30°C for 24 h. The methods of isolation of planktonic strains and identification of all the isolates using Sanger sequencing were the same as previously described (Zheng et al., 2012).

### Formation and Structural Observations of Biofilms

A scanning electron microscope (SEM; Hitachi, model SU8010, Japan) was used to examine the biofilm structure in the *Daqu* sample and the solid-state co-culture samples.

Biofilm formation of the isolates was analyzed in flat-bottomed 96-well polystyrene microtiter plates using the crystal violet assay. To evaluate the biofilm formation ability of isolates, isolates were diluted to 10^6^ cfu/mL by using MRS for LAB and YPD for yeasts. And wells were filled with 200 μL of diluted pure cultures.

To determine the interaction pattern, LAB was diluted to 10^6^ cfu/mL and yeast was diluted to 10^4^ cfu/mL in liquid *Daqu* media. For the dual-species biofilm, 100 μL of each diluted culture were mixed then added to each well. For single-species control, 100 μL diluted culture and 100 μL media were added into each well. Then the plate was incubated for 3 days at 30°C under static conditions. The biomass was measured after staining with 1% w/v crystal violet and measuring the optical absorbance at 595 nm. Each experiment was repeated in triplicate.

### Enzyme Activities Assay and Fortified Fermentation

The measurements of enzyme activities were similar to those previously described (Liu et al., 2018) with minor modifications. The temperature used for all activity assays was 30°C. One unit of starch amylolytic enzymatic activity was defined as the amount of enzyme required to release 1 μmol of reducing sugars (with glucose as a standard) per mL of suspension per minute. One unit (U) of protease activity was defined as the amount of enzyme required to release 1 μg of tyrosine per mL of suspension per minute. 4-Nitrophenyl acetate was used as a substrate to determine esterase activity. One unit of enzymatic activity was defined as the amount of enzyme required to liberate 1 μmol of 4-nitrophenyl acetate per mL of suspension per minute.

The methods of fortified fermentation were the same as those previously described (Li et al., 2018). Headspace solid-phase microextraction coupled with gas chromatography-mass spectrometry (HS-SPME-GC-MS) was used to analyze volatile compounds in fermented grains (Pang et al., 2018). For each sample, 2 g was mixed with 8 mL of Milli-Q water in triplicate. After 30 min of ultrasonic treatment, the sample solution was centrifuged at 8,000 × *g* for 10 min at 4°C. Then, 8 mL of the supernatant, 3 g sodium chloride, and 2 μL of the internal standard (4-methyl-2-pentanol, 125.0 mg/L) were added to a 20 mL vial. Volatile compounds from the sample were collected in an SPME fiber (50:30 mm divinylbenzene–carboxy–polydimethylsiloxane, DVB/CAR/PDMS; Supelco Co., Bellefonte, PA, United States) at 50°C for 5 min and extracted for 45 min. Then, the fiber was inserted into the GC-MS analyzer and the volatile compounds were identified by comparing their mass spectra with those in the NIST 14 mass spectral database. The content of each compound was calculated by comparison of its spectral peak area with the internal standard (4-methyl-2-pentanol).

### Detection of Volatile and Non-volatile Compounds in Solid-State Co-culture

To simulate the original environment of the starter community, a mixture of 6 g barley powder, 4 g pea powder, and 5 mL distilled water was used as the solid medium for co-culture. The medium was sterilized by autoclaving at a temperature of 121°C under 15 psi pressure for 15 min. LAB and yeast were mixed in barley and pea liquid media with a ratio of 100:1 (LAB strains were diluted to 10^6^ cfu/mL and yeast 10^4^ cfu/mL). After sterilization, 1 mL of mixed suspension was added to solid media and incubated for 7 days at 30°C under static conditions. The control was inoculated without microorganisms, and the single-species control was inoculated with a suspension of only a single strain (as the same dilution used in the co-culture). Each experiment was repeated triplicate. After incubation, 85 mL PBS buffer was added to the co-culture system. Serial dilutions were prepared from this suspension and LAB were enumerated on MRSA with 0.1% w/v natamycin to prevent yeast growth for 3 days at 30°C. Yeasts were enumerated on YPD agar with 100 μg/mL chloramphenicol to prevent LAB growth for 3 days at 30°C.

Non-volatile compounds in solid-state co-culture were analyzed using ^1^H NMR spectroscopy as previously described (Zheng et al., 2011a) with minor modifications of sample preparation. Samples (0.5 g) were homogenized with 1.5 mL ultra-pure water for 60 s in a mini-bead beater then placed on ice for 10 min. Mixtures were centrifuged for 10 min at 10,000 × *g* and 4°C and the supernatants retained. To each supernatant, 1 mL of 0.2 mM phosphate buffer (pH 7.0) containing 20% w/w of deuterium oxide, 1 mM 3-trimethylsilyl-2,2,3,3-d4-propionate (TSP), 10 mM imidazole and 0.2% w/w sodium azide was added and mixed thoroughly. The mixtures were centrifuged at 10,000 × *g* and 4°C for 10 min and the supernatants were transferred into NMR tubes for ^1^H NMR analysis at 300 K using an Avance III 600 MHz spectrometer (Bruker, Karlsruhe, Germany) fitted with a 5 mm PATXI probe. Non-volatile metabolites were identified and quantified using the Chenomx software (version 5.0; Chenomx, Edmonton, AB, Canada) and trimethylsilylpropanoic acid as an internal reference. Volatile compounds in solid-state co-culture were analyzed using the same methods applied to fermented grains of fortified fermentation.

### Statistical Analysis of Data

The SPSS V22.0 software was used to determine differences between microbial enumeration in pure culture and co-culture using one-way analysis of variance. Metabolites produced during solid-state incubation were analyzed using principal component analysis (PCA) using Clutvis (Metsalu and Vilo, 2015). Differential metabolites were identified using partial least squares discriminant analysis (PLS-DA) with variable important on projection (VIP) larger than 1 and *p* > 0.05. The data were standardized to present the changes relative to the control group, and heatmaps of differential metabolites were constructed using the pheatmap package in R.

## Results

### Microbial Diversity in TAFS Revealed by Metagenomics

**FIGURE 1:**
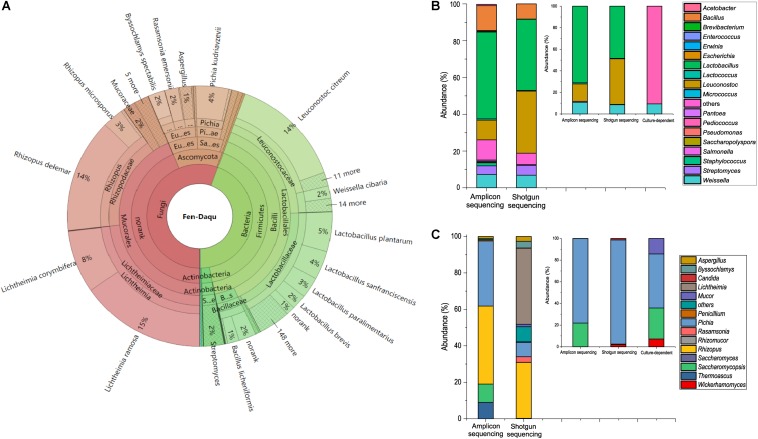
Taxonomic profile of the starter community. Shotgun metagenomic analysis of community structure visualized using KRONA **(A)**. Comparison of bacterial diversity **(B)** and fungal diversity **(C)** at the genus level analyzed using the amplicon sequencing and shotgun metagenomic sequencing method. Especially, diversity of LAB **(B)** and yeasts **(C)** at the genus level analyzed using culture-dependent method and culture-independent methods in the small plots.

To investigate the existence of functional microorganisms in a microbial community, we performed amplicon sequencing and shotgun metagenomic sequencing to investigate the complex community structure of TAFS. PCR-based amplicon sequencing was used to analyze the bacterial and fungal diversity of *Daqu* (Figure 1). A total of 5 bacterial phyla and 43 genera were identified. Firmicutes were the main bacterial phylum with an abundance of over 89% in the community. The dominant bacterial genus was *Lactobacillus* followed by *Leuconostoc*, *Bacillus*, *Weissella*, and *Streptomyces*. Three fungal phyla and 13 genera were found using amplicon sequencing. Ascomycota and Zygomycota were the major fungal phyla. *Rhizopus* was the dominant fungal genus, followed by *Pichia*, *Saccharomycopsis*, *Thermoscus*, and *Aspergillus*.

A total of 97338490 raw reads were detected by shotgun metagenomic sequencing. The statistics of shotgun data were presented in the Supplementary Tables S1, S2. The results of shotgun metagenomic taxonomic composition were explored using the Krona visualization tool (Figure 1A). The composition of the *Daqu* microbial community was 55.71% fungal, 44.28% bacterial, and 0.0045% archaeal. A total of 1847 species from 781 genera were detected by shotgun metagenomics. Moreover, LAB accounted for up to 36% of the microbial abundance. Zygomycota was the dominant phylum, followed by Firmicutes and Ascomycota. Eleven dominant genera were found with an abundance of over 1%. *Lichtheimia*, *Lactobacillus*, *Rhizopus*, and *Leuconostoc* were extremely abundant, comprising 23%, 18%, 17%, and 16% of the microbial population, respectively. There were 15 dominant species with an abundance over 1%. *Lichtheimia ramose*, *Rhizopus delemar*, *Leuconostoc citreum*, *Lichtheimia corymbifera*, and *L. plantarum* comprised 15, 14, 14, 8, and 5% of the microbial population, respectively. Although alcoholic fermentation starter was a complex community with 781 genera, the total abundance of the 11 dominant genera was 93.05%. The alcoholic fermentation starter therefore appears to be a diverse but relatively concentrated microbial community.

To investigate whether functional species could be uncultivated, isolation and identification of *Lactobacillus* and *S. cerevisiae* were performed with MRSA and YPD media. A total of 32 strains were isolated by MRSA including 29 *Pediococcus* strains and 3 *Weissella* strains (Supplementary Table S3). And 14 yeast strains were isolated, including *Pichia kudriavzevii*, *Pichia anomalus*, and *Saccharomycopsis fibuligera* (Supplementary Table S4). Analysis of the combined microbial diversity data obtained using culture-dependent methods and metagenomic methods, showed that *S. cerevisiae* and *Lactobacillus* sp. were detected only by culture-independent methods. *S. cerevisiae* was only detected by shotgun sequencing with an abundance of 0.005% (4807 reads) in the community. Both metagenomic methods indicated that *Lactobacillus* was one of the dominant genera in the community. A total of 153 species within the genus *Lactobacillus* were detected by shotgun sequencing, which makes *Lactobacillus* the most diverse genus in the community. *L. plantarum*, *Lactobacillus sanfranciscensis*, *Lactobacillus paralimentarius*, *Lactobacillus brevis* were the dominant species covered 5%, 4%, 3%, and 2%, respectively in the community. *S. cerevisiae* and *Lactobacillus* sp. were identified as unculturable species in alcoholic fermentation starter community.

### Mixed Biofilm Exists in TAFS

**FIGURE 2:**
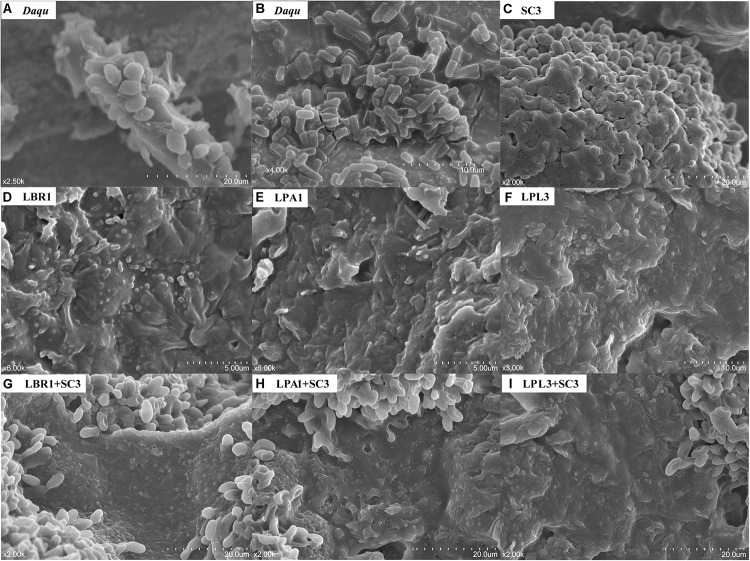
Scanning Electron Microscope (SEM) images of biofilms. Mixed biofilm within the alcoholic starter community **(A,B)**. Pure culture of *S. cerevisiae* **(C)** and *Lactobacillus* sp. **(D–F)** on solid media. Co-culture of *S. cerevisiae* and *Lactobacillus* sp. **(G–I)** on solid media. LBR, *L. brevis*; LPA, *L. paralimentarius*; LPL, *L. plantarum*; SC, *S. cerevisiae*.

Based on the identification of unculturable species in TAFS, we hypothesized that mixed biofilm might be their strategy to survive in the community. To test whether the formation of a mixed biofilm represents a survival strategy for unculturable *S. cerevisiae* and *Lactobacillus* sp., we examined the mixed biofilm formed by diverse microorganisms in the *Daqu* sample using SEM (Figures 2A,B and Supplementary Figure S2). The samples were directly coated with a gold layer without any treatment to preserve the original condition of the microorganisms. The mixed biofilm in *Daqu* comprised a mature biofilm with thick layers of EPS, suggesting the existence of efficient biofilm producers in the community. Based on the size and shape, yeasts as well as bacteria such as bacilli and cocci were the likely producers of various mixed biofilms in the *Daqu* sample.

Compared with planktonic cells, once microorganisms adhere to the surface and form the biofilm, it is difficult to isolate them from serial dilutions with standard media. However, the potential biofilm producers can be predicted by analyzing the data obtained from culture-dependent and culture-independent assays of microbial diversity. Several dominant genera in *Daqu* including *Bacillus*, *Pichia*, *Saccharomycopsis*, *Lichtheimia*, *Aspergillus*, and *Rhizopus* have previously been detected by culture-dependent methods (Zheng et al., 2012). *S. cerevisiae* and *Lactobacillus* sp. were only detected by culture-independent methods. Based on the biofilm hypothesis, *S. cerevisiae* and *Lactobacillus* sp. might be the potential biofilm producers in the community.

### *S. cerevisiae* and *Lactobacillus* sp. Are Biofilm Producers

To provide further evidence that mixed biofilm formation is a survival strategy for certain microorganisms, in particular to identify the biofilm producers, species growing in the biofilm state were isolated and identified. *Daqu* media with barley and pea was used to simulate the original environment of the community, and microbial diversity was reduced through incubation under anaerobic environment for 7 days for biofilm dispersal. A total of 44 isolates including 24 bacterial and 20 fungal strains were retrieved from the *Daqu* community and identified using 16S/28S rRNA sequencing (Supplementary Tables S5, S6). All of the bacterial strains were *Lactobacillus* sp. and 19 out of the 20 fungal strains were *S. cerevisiae*. Within the genus *Lactobacillus*, *L. paralimentarius* was the dominant species, followed by *L. brevis* and *L. plantarum*. The observed diversity of the genus *Lactobacillus* was also concordant with the findings from metagenomic sequencing.

**FIGURE 3:**
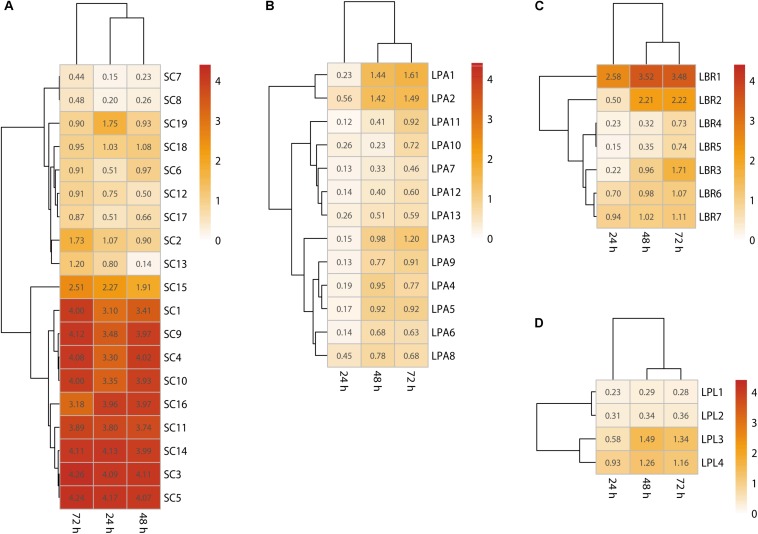
Single-species biofilms formed by *S. cerevisiae* **(A)**, *L. paralimentarius* **(B)**, *L. brevis* **(C)**, and *L. plantarum* **(D)**. LBR, *L. brevis*; LPA, *L. paralimentarius*; LPL, *L. plantarum*; SC, *S. cerevisiae*.

To identify the main biofilm producers in the community, biofilm formation ability of each isolate was assessed using crystal violet staining. The results showed most *Lactobacillus* sp. could form a biofilm after 72 h (Figure 3), with adherence requiring more than 24 h and the formation of a mature biofilm requiring 48–72 h. In particular, the optical density of the strongest biofilm formed by the *L. brevis*, *L. plantarum*, and *L. paralimentarius* strains after 72 h was 3.48, 1.61, and 1.34 at 595 nm, respectively. Thus, *L. brevis* was the strongest biofilm producer among all the bacterial isolates. For fungal isolates, most *S. cerevisiae* strains could form a mature biofilm (Figure 3), requiring less than 24 h for adherence and 48 h for the formation of the mature biofilm. The highest optical density of biofilm formed by *S. cerevisiae* was 4.13 after 48 h and 4.11 after 72 h (Figure 3). In order to eliminate the effects of media, biofilm formation ability of isolates was also tested with *Daqu* media using the same method, the results were similar (Supplementary Figure S1). These results confirmed that *S. cerevisiae* and *Lactobacillus* sp. survived in this community as biofilm producers, while the faster biofilm formation and greater biomass production of *S. cerevisiae* indicated it was the dominant biofilm producer.

### Biofilm Producers Are Also Functional Microbes in Alcoholic Fermentation

**FIGURE 4:**
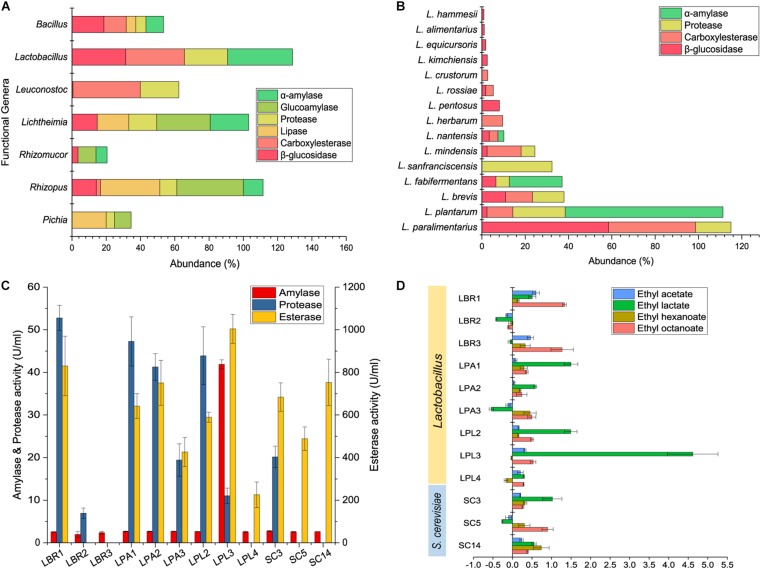
Function prediction and validation of biofilm producers. KEGG annotation of functional genera **(A)** and *Lactobacillus* sp. **(B)** in starter community. Hydrolase activities of *Lactobacillus* sp. and *S. cerevisiae* **(C)**. Analysis of the main flavor compounds produced during fortified fermentation with *Lactobacillus* sp. and *S. cerevisiae* **(D)**. LBR, *L. brevis*; LPA, *L. paralimentarius*; LPL, *L. plantarum*; SC, *S. cerevisiae*.

Kyoto Encyclopedia of Genes and Genomes annotation was conducted using the shotgun metagenomic sequencing data to predict the functional genera and the functions of biofilm producers in the community. Genes related to five representative enzymes were annotated using the KEGG database to reveal the functional genera. α-Amylase, glucoamylase, and protease are involved in the degradation of raw materials within the alcoholic fermentation starter, while lipase, carboxylesterase, and β-glucosidase play a role in flavor development during alcoholic fermentation. *Lactobacillus*, *Rhizopus*, *Lichtheimia*, *Leuconostoc*, *Bacillus*, *Pichia*, and *Rhizomucor* were the functional genera at the gene level (Figure 4A), with *Lactobacillus* being the major functional genera in the community. Since *Lactobacillus* is a diverse genus, functional analysis was conducted to reveal the functional *Lactobacillus* species. Fifteen were annotated with potential functions (Figure 4B), with *L. paralimentarius*, *L. plantarum*, and *L. brevis* being identified as the core functional species. A potential role was identified for *L. plantarum* in starch degradation and for *L. paralimentarius* and *L. brevis* in flavor development.

To validate the functions of biofilm producers and to select functional strains for further interaction studies, three representative strains of each species with strong biofilm-forming ability were screened for enzyme production. Enzyme activity assays showed that all could produce esterase, with enzyme activities above 200 U/mL, some *Lactobacillus* strains could produce protease, while only one *L. plantarum* strain produced high levels of amylase (Figure 4C). Discernable differences were observed among different strains of the same species. Particularly, LPL3 was capable of producing high levels of amylase, esterase, and protease with the enzyme activities of 41.90, 11.04, and 1004.39 U/mL, respectively. LBR1 was the major enzyme producer among the *L. brevis* strains, and SC3 was the only *S. cerevisiae* strain able to produce protease.

Based on enzyme activities, the major function of biofilm producers might be the synthesis of flavor substances composed mainly of esters. We detected major esters including ethyl acetate, ethyl lactate, ethyl hexanoate, and ethyl octanoate in fermented grains, and analyzed the relative rate of their changes in concentration after fortification with biofilm producers (Figure 4D). All of the selected strains could increase the concentration of at least two major esters, except for the *L. brevis* strain LBR2. Diverse *Lactobacillus* species imparted various effects on the community. Adding *L. brevis* to the community could increase the concentration of ethyl octanoate. Fortified fermentation with *L. plantarum* and *L. paralimentarius* could drive the community to produce more ethyl lactate. Combining the results of biofilm formation and fortified fermentation, *L. brevis* strain LBR1, *L. plantarum* strain LPL3, *L. paralimentarius* strain LPA1, and *S. cerevisiae* strain SC3 were selected as representatives of each species for further investigation.

### Microbial Interactions Among Biofilm Producers

**FIGURE 5:**
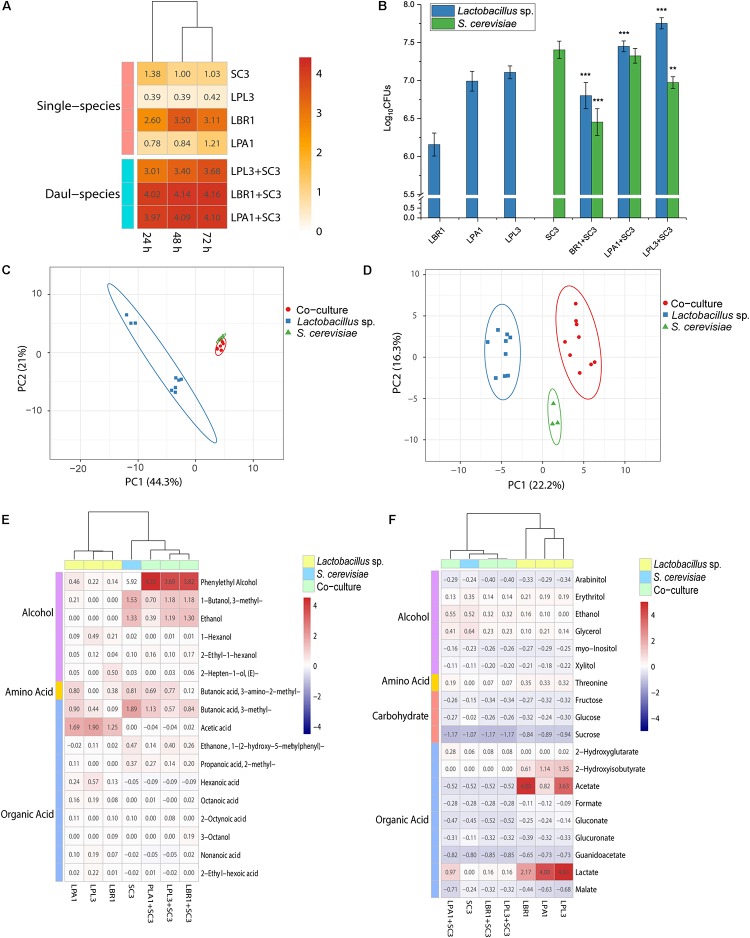
Interactions between biofilm producers. Biomass of the mixed biofilm formed by *Lactobacillus* sp. and *S. cerevisiae* in liquid media **(A)** and solid media **(B)**. **p < 0.01; ***p < 0.001. Principal component analysis of non-volatile **(C)** and volatile **(D)** metabolites in the solid-state co-culture of biofilm producers. Heatmap of differential non-volatile metabolites **(E)** and volatile metabolites **(F)** in the solid-state co-culture of biofilm producers.

To investigate the interaction patterns between *Lactobacillus* sp. and *S. cerevisiae*, the biomass of single-species and mixed-species biofilms were measured in the liquid medium (Figure 5A). The strains need to survive by deriving nutrition from barley and pea (the raw materials for making the starter). Among all the selected strains, *L. brevis* showed the strongest ability to form a biofilm in the *Daqu* medium. The biomass of *Lactobacillus* sp. and *S. cerevisiae* co-culture was much higher than the biomass of two single strains and even higher than the sum of the biomass of two single species. In the mixed-species biofilm, *Lactobacillus* sp. and *S. cerevisiae* produced more EPS to survive through mutualism.

Since the alcoholic fermentation starter is made using solid-state fermentation, the growth and metabolic analyses were performed in the solid state with smashed barley and pea. Although co-culture in the liquid state demonstrated mutualism between *Lactobacillus* sp. and *S. cerevisiae* to produce a biofilm, the plate-counting method showed that the growth of *Lactobacillus* sp. was significantly promoted during co-culture with *S. cerevisiae* in the solid state (Figure 5B). The growth of *S. cerevisiae* was significantly inhibited during co-culture with *L. brevis* and *L. plantarum*. *L. brevis* had the strongest inhibitory effect on the growth of *S. cerevisiae*, while the growth of all the *Lactobacillus* strains was significantly promoted by co-culture with *S. cerevisiae*. Living in the mixed-species biofilm with *S. cerevisiae* could increase the cell survival of *Lactobacillus* in the alcoholic fermentation starter community.

To reveal the interaction mechanism in the solid state, the structure of the mixed-species biofilm was analyzed using SEM (Figure 2). Both single-species and mixed-species samples were observed directly. The cell content of *Lactobacillus* sp. was lower in single- versus mixed-species samples, while the opposite was true for *S. cerevisiae*. Only *L. brevis* and *S. cerevisiae* could form a single-species biofilm on the surface of barley and pea powders. Mixed-species biofilms existed in each co-culture sample with the same two-layer spatial structure. In the mixed-species biofilm, the bottom layer consisted of *Lactobacillus* sp. and *S. cerevisiae* occupied the upper layer.

In addition to growth analysis, the metabolites of single-species and co-culture samples were analyzed using ^1^H-NMR and GC-MS. The non-volatile metabolites of co-culture were clustered with *S. cerevisiae* in the PCA plot (Figure 5C), with ethanol and glycerol being the typical metabolites. *Lactobacillus* strains were clustered together in the PCA plot based on acetate and lactate as the typical metabolites. Nineteen differential non-volatile metabolites were analyzed using PLS-DA, and the concentrations of typical metabolites were decreased in co-culture samples (Figure 5E). The concentrations of fructose and glucose in co-culture were similar those measured in *Lactobacillus* sp. mono-culture. *L. brevis* and *L. plantarum* lost the ability to produce lactate, acetate, and 2-hydroxyisobutyrate in the mixed-species biofilm. During co-culture in solid media, *Lactobacillus* sp. had extremely low metabolic activity and *S. cerevisiae* mainly contributed to non-volatile metabolites in the mixed-species biofilm.

Analysis of the volatile metabolites revealed patterns similar to those exhibited by the non-volatile metabolites. *S. cerevisiae* was clustered with co-culture samples in the PCA plot while *Lactobacillus* sp. mono-culture samples were clustered together (Figure 5D). Phenylethyl alcohol, 3-methyl-butanoic acid, 3-methyl-1-butanol, and ethanol were typical volatile metabolites of *S. cerevisiae*, while acetate and 2,4-di-tert-butylphenol were typical of the *Lactobacillus* strains. More specifically, hexanoic acid, 1-hexanol, and 2-heptenal were typical metabolites of *L. paralimentarius*, and 2-hepten-1-ol was typical of *L. brevis*. The analysis of 24 differential compounds using PLS-DA showed that the concentrations of typical metabolites in co-culture samples were decreased (Figure 5F). During co-culture with *S. cerevisiae*, the metabolic activity of *Lactobacillus* sp. was significantly inhibited and *S. cerevisiae* was the main source of non-volatile metabolites in the mixed-species biofilm.

## Discussion

This study demonstrated that formation of a mixed biofilm is a survival strategy for unculturable microorganisms in a complex microbial community. Our work revealed the existence of *S. cerevisiae* and *Lactobacillus* sp. in traditional Chinese alcoholic fermentation starter and these species were predominantly responsible for the formation of single-species and mixed-species biofilms. These biofilm-producing cells also expressed various hydrolases that generate ethanol and flavor compounds during alcoholic fermentation. For *Lactobacillus* strains, their interaction with *S. cerevisiae* in a mixed biofilm increased their biomass and reduced their metabolic activity. In contrast, *S. cerevisiae* predominated the metabolic activity despite having a slightly decreased biomass in the mixed biofilm. With limited nutrients in the starter community, *Lactobacillus* strains tended to cover the bottom layer while *S. cerevisiae* occupied the top layer with planktonic cells. The presence of a mixed biofilm in the starter community can be exploited in future studies to address unclear questions about fermentation mechanisms, succession, and reconstruction of the starter community. Formation of a mixed biofilm could be the explanation for unculturable microorganisms in other complex environmental microbial communities.

Several differences in the community composition could be identified from a comparison of the isolation, amplicon sequencing, and shotgun metagenomic sequencing data, which is common in community analyses of spontaneous fermentation and fermentation starters such as cocoa and vinegar (Li et al., 2014, 2015; De Vuyst and Weckx, 2016). The selective bias caused by ITS primers was the main reason for the differences between fungal diversity results of amplicon and shotgun sequencing (Toju et al., 2012; Quast et al., 2013). Moreover, the observation and isolation of a mixed biofilm in the starter community can explain the differences observed using culture-dependent versus culture-independent methods. Microorganisms in a mature biofilm can be difficult to isolate and cultivate. To isolate the unculturable LAB and *S. cerevisiae*, we therefore adjusted the incubation conditions to reduce the number and diversity of microorganisms within the multi-species fermentation starter before cultivation. Additionally, we used a specific medium of barley and pea (the raw materials of alcoholic fermentation starter) to simulate the original natural environment of unculturable species *in vitro*. Biofilms provide a microbial lifestyle that allows microorganisms to survive or thrive in nutrient-poor environments (Vartoukian et al., 2010). There exist few nutrients in the fermentation starter, with barley and pea providing mainly starch, making it difficult for *Lactobacillus* sp. and *S. cerevisiae* strains with low amylase activity to survive as planktonic cells. Due to their co-existence with strong amylase producers such as *Bacillus* and *Rhizopus*, *Lactobacillus* sp. and *S. cerevisiae* formed a mixed-species biofilm. The increased biomass of *Lactobacillus* sp. and *S. cerevisiae* co-culture in this environment is consistent with assertions that biofilm is a response to ecological competition (Oliveira et al., 2015). Biofilm formed by *S. cerevisiae* in wine fermentation is an adaptive mechanism for continued growth on non-fermentable ethanol (Zara et al., 2010). And *S. cerevisiae* strains also display biofilm-like morphology as an adaptive mechanism for polyphenols from grapes and wine (Sidari et al., 2014). Thus, mixed biofilm could be a survival strategy not only for species in a fermentation starter but also for other unculturable species in complex microbial communities.

We identified protease and esterase activities associated with *Lactobacillus* sp. and *S. cerevisiae* in mixed biofilm, suggesting that they could produce flavor compounds, especially esters, during fermentation. The increased number of esters produced during fortified fermentation with biofilm isolates also proved their existence was a positive disturbance to microbial community and that is beneficial for alcoholic fermentation. Previous microbial diversity analyses during alcoholic fermentation reported that *Lactobacillus* sp. and *S. cerevisiae* were the dominant microorganisms related to flavor development (Pang et al., 2018). Consistent with most fermented foods and starters, the functional species in TAFS were *Lactobacillus* sp. and *S. cerevisiae* (Tamang et al., 2016). Also, the research of fermented cabbage explained that although LAB is hardly detected in the Napa cabbage phyllosphere, the anaerobic, salty, and sugar-rich conditions of the fermentation environment make them dominant in the cabbage fermentation (Miller et al., 2019). Similar to cabbage fermentation, the suitable fermentation environment could be the reason for the dominance of *Lactobacillus* sp. and *S. cerevisiae* in alcoholic fermentation. The similar functions of *Lactobacillus* sp. and *S. cerevisiae* in the starter community and during alcoholic fermentation underline that the starter could be the source of the functional species during alcoholic fermentation. Based on the results, *Lactobacillus* sp. and *S. cerevisiae* formed mixed biofilm in the fermentation starter. Under sufficient nutrition and culture conditions, the amount of *Lactobacillus* sp. and *S. cerevisiae* were increased through biofilm dispersal. Microbial succession of alcoholic fermentation showed that *Bacillus*, *Rhizopus*, *Pichia*, and *Saccharomycopsis* were the predominant genera in the beginning of alcoholic fermentation and their abundance decreased as fermentation processed (Li et al., 2017; Pang et al., 2018). *Lactobacillus* sp. and *S. cerevisiae* were shown to become the dominant genus and molds died out after 7 days of alcoholic fermentation based on the results of amplicon sequencing (Jin et al., 2017; Pang et al., 2018). And *Lactobacillus acetotolerans* is the only dominant species in the last stage of alcoholic fermentation (Pang et al., 2018). Therefore, we hypothesize that alcoholic fermentation in the solid state is divided into three stages. In the first stage, aerobic microorganisms degrade starch by producing amylases, while *Lactobacillus* sp. and *S. cerevisiae* survive in a mixed-species biofilm without dispersal. In the second stage, aerobic microorganisms die due to limited oxygen. With suitable nutrients such as glucose, active dispersal of *Lactobacillus* sp. and *S. cerevisiae* spread from the mixed biofilm to produce ethanol and flavor compounds. In the last stage, most species die because of the accumulation of alcohol and acidity during the alcoholic fermentation, and only microorganisms with high tolerance survive. The results that *L. plantarum*, *L. brevis* were the dominant species in genus *Lactobacillus* at 7, 15 days of alcoholic fermentation (Li et al., 2017), while they were the biofilm producers in starter community, which also support our hypothesis. The diversity of genus *Lactobacillus* was decreased at the end of alcoholic fermentation, and only *L. acetotolerans* could survive (Pang et al., 2018). Therefore, the core of TAFS could be a solid-state vector comprising a mixed-species biofilm of *Lactobacillus* sp. and *S. cerevisiae* along with starch-degrading microorganisms such as *Bacillus* sp. and molds.

**FIGURE 6:**
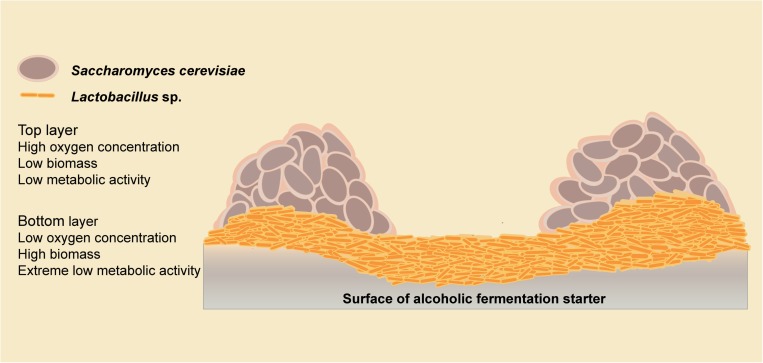
Model of microbial interactions among the biofilm producers.

We have demonstrated microbial interaction patterns among biofilm isolates using biomass and metabolite analyses. Mixed-species biofilms with high biodiversity showed better resilience in response to environmental disturbance (Feng et al., 2017), which might be the result of microbial interactions. In the starter community, the environmental adaptability of *Lactobacillus* strains was improved, and more cells survived in a mixed-species biofilm than in the planktonic state. Meanwhile, the metabolic activity of all the species was decreased, which could be explained using the spatial interaction hypothesis. Spatial interactions are widespread in a mixed-species biofilm are enable significant important community functions (Nadell et al., 2016). We propose a double-layer spatial model in this mixed-species biofilm (Figure 6), with *Lactobacillus* strains residing at the bottom in a low oxygen environment, resulting in a low metabolic activity. The biomass of *S. cerevisiae* strains was slightly decreased because of the competition with *Lactobacillus* strains. With greater access to the oxygen, *S. cerevisiae* strains in the top layer of the biofilm could maintain a higher level of metabolic activity. Thus, the metabolic fingerprints of solid co-culture are similar to *S. cerevisiae* strains. Studies of a double-layer mixed biofilm comprising *L. plantarum* and *S. cerevisiae* on a glass surface also support this double-layer spatial structure (Urukawa et al., 2010). The microbial interactions among *Lactobacillus* sp. and *S. cerevisiae* are responsible for forming the proposed double-layer structure of the mixed biofilm.

## Conclusion

In conclusion, *Lactobacillus* sp. and *S. cerevisiae* with the function of producing ethanol and flavor compounds, formed a mixed-species biofilm to survive in starter community while co-existing with other microorganisms. With culturable *Lactobacillus* sp. and *S. cerevisiae* isolated from the community, all of the dominant species in TAFS could be cultivated. The starter community therefore represents a new cultivation-based model system that can be used to explore fundamental aspects of microbial ecology. Identification of the existence and function of biofilm producers also increases the possibility of community reconstruction, which could lead to industrialization of Baijiu production and provide a more consistent quality of the alcoholic fermentation starter. Future studies of microbial interactions between species in biofilm-state and planktonic species could be crucial to rebuild the starter community *in vitro*.

## Data Availability Statement

The datasets generated for this study can be found in the Sequence Read Archive (SRA) at the NCBI under the project accession number PRJNA587574.

## Author Contributions

YF designed and conducted the experimental work assisted by XH. YF and XH performed the data analysis. YF wrote the first draft of the manuscript. XH and JC contributed to the manuscript revision. BH contributed to the supervision, manuscript revision, and overall support of this study. All authors read and approved the final version of the manuscript.

## Conflict of Interest

The authors declare that the research was conducted in the absence of any commercial or financial relationships that could be construed as a potential conflict of interest.
