# Supplementary material for: Formation of a Mixed-Species Biofilm Is a Survival Strategy for Unculturable Lactic Acid Bacteria and Saccharomyces cerevisiae in Daqu, a Chinese Traditional Fermentation Starter
Source: Front Microbiol. 2020 Feb 6;11:138. doi: 10.3389/fmicb.2020.00138 (PMC7015947; doi:10.3389/fmicb.2020.00138)
Supplement: Supplementary file 1 [file Data_Sheet_1.docx]

Supplementary Material

# Supplementary Figures and Tables

Table S1. Statistics of shotgun metagenomic sequencing (raw data)

| Read length  (bp) | Raw reads | Raw bases  (bp) |
| --- | --- | --- |
| 150 | 97338490 | 14698111990 |

Table S2. Statistics of shotgun metagenomic sequencing (clean data)

| Clean reads | Clean bases (bp) | Percent in raw reads (%) | Percent in raw bases  (%) |
| --- | --- | --- | --- |
| 96139787 | 14368263368 | 98.77 | 97.76 |

Table S3. Lactic acid bacteria isolated from *Daqu*

| Code | Species identification | Accession No. | Identity (%) |
| --- | --- | --- | --- |
| L1 | *Pediococcus pentosaceus* | MN638773 | 100 |
| L2 | *Pediococcus pentosaceus* | MN638774 | 100 |
| L3 | *Weissella cibaria* | MN638775 | 99 |
| L4 | *Pediococcus pentosaceus* | MN638776 | 99 |
| L5 | *Pediococcus lolii* | MN638777 | 100 |
| L6 | *Pediococcus pentosaceus* | MN638778 | 99 |
| L7 | *Pediococcus lolii* | MN638779 | 100 |
| L8 | *Pediococcus acidilactici* | MN638780 | 99 |
| L9 | *Pediococcus lolii* | MN638781 | 100 |
| L10 | *Pediococcus pentosaceus* | MN638782 | 100 |
| L11 | *Weissella cibaria* | MN638783 | 100 |
| L12 | *Pediococcus pentosaceus* | MN638784 | 100 |
| L13 | *Pediococcus pentosaceus* | MN638785 | 99 |
| L14 | *Pediococcus lolii* | MN638786 | 100 |
| L15 | *Pediococcus pentosaceus* | MN638787 | 99 |
| L16 | *Pediococcus pentosaceus* | MN638788 | 100 |
| L17 | *Pediococcus pentosaceus* | MN638789 | 99 |
| L18 | *Pediococcus pentosaceus* | MN638790 | 99 |
| L19 | *Weissella paramesenteroides* | MN638791 | 99 |
| L20 | *Pediococcus lolii* | MN638792 | 100 |
| L21 | *Pediococcus pentosaceus* | MN638793 | 100 |
| L22 | *Pediococcus pentosaceus* | MN638794 | 100 |
| L23 | *Pediococcus lolii* | MN638795 | 100 |
| L24 | *Pediococcus pentosaceus* | MN638796 | 100 |
| L25 | *Pediococcus pentosaceus* | MN638797 | 100 |
| L26 | *Pediococcus acidilactici* | MN638798 | 99 |
| L27 | *Pediococcus pentosaceus* | MN638799 | 100 |
| L28 | *Pediococcus pentosaceus* | MN638800 | 100 |
| L29 | *Pediococcus pentosaceus* | MN638801 | 99 |
| L30 | *Pediococcus pentosaceus* | MN638802 | 99 |
| L31 | *Pediococcus pentosaceus* | MN638803 | 100 |
| L32 | *Pediococcus lolii* | MN638804 | 99 |

Table S4. Yeasts isolated from *Daqu*

| Code | Species identification | Accession No. | | Identity (%) |
| --- | --- | --- | --- | --- |
| Y1 | *Pichia kudriavzevii* | MN648835 | 100 | |
| Y2 | *Wickerhamomyces anomalus* | MN648836 | 100 | |
| Y3 | *Pichia kudriavzevii* | MN648837 | 100 | |
| Y4 | *Pichia kudriavzevii* | MN648838 | 99 | |
| Y5 | *Pichia kudriavzevii* | MN648839 | 100 | |
| Y6 | *Pichia kudriavzevii* | MN648840 | 100 | |
| Y7 | *Issatchenkia orientalis* | MN648841 | 100 | |
| Y8 | *Clavispora lusitaniae* | MN648842 | 99 | |
| Y9 | *Pichia kudriavzevii* | MN648843 | 100 | |
| Y10 | *Pichia kudriavzevii* | MN648844 | 100 | |
| Y11 | *Saccharomycopsis fibuligera* | MN648845 | 100 | |
| Y12 | *Saccharomycopsis fibuligera* | MN648846 | 100 | |
| Y13 | *Saccharomycopsis fibuligera* | MN648847 | 100 | |
| Y14 | *Saccharomycopsis fibuligera* | MN648848 | 100 | |

Table S5. Lactic acid bacteria isolated from biofilm in *Daqu*

| Code | Species identification | Accession No. | Identity (%) |
| --- | --- | --- | --- |
| LBR1 | *Lactobacillus brevis* | MN640540 | 100 |
| LBR2 | *Lactobacillus brevis* | MN640541 | 100 |
| LBR3 | *Lactobacillus brevis* | MN640542 | 100 |
| LBR4 | *Lactobacillus brevis* | MN640543 | 100 |
| LBR5 | *Lactobacillus brevis* | MN640544 | 100 |
| LBR6 | *Lactobacillus brevis* | MN640545 | 100 |
| LBR7 | *Lactobacillus brevis* | MN640546 | 99 |
| LPA1 | *Lactobacillus paralimentarius* | MN640547 | 99 |
| LPA10 | *Lactobacillus paralimentarius* | MN640548 | 99 |
| LPA11 | *Lactobacillus paralimentarius* | MN640549 | 100 |
| LPA12 | *Lactobacillus paralimentarius* | MN640550 | 99 |
| LPA13 | *Lactobacillus paralimentarius* | MN640551 | 99 |
| LPA2 | *Lactobacillus paralimentarius* | MN640552 | 99 |
| LPA3 | *Lactobacillus paralimentarius* | MN640553 | 100 |
| LPA4 | *Lactobacillus paralimentarius* | MN640554 | 99 |
| LPA5 | *Lactobacillus paralimentarius* | MN640555 | 99 |
| LPA6 | *Lactobacillus paralimentarius* | MN640556 | 99 |
| LPA7 | *Lactobacillus paralimentarius* | MN640557 | 99 |
| LPA8 | *Lactobacillus paralimentarius* | MN640558 | 99 |
| LPA9 | *Lactobacillus paralimentarius* | MN640559 | 99 |
| LPL1 | *Lactobacillus plantarum* | MN640560 | 99 |
| LPL2 | *Lactobacillus plantarum* | MN640561 | 99 |
| LPL3 | *Lactobacillus plantarum* | MN640562 | 100 |
| LPL4 | *Lactobacillus plantarum* | MN640563 | 99 |

Table S6. Yeasts isolated from biofilm in *Daqu*

| Code | Species identification | Accession No. | Identity (%) |
| --- | --- | --- | --- |
| SC1 | *Saccharomyces cerevisiae* | MN648815 | 100 |
| SC2 | *Saccharomyces cerevisiae* | MN648816 | 100 |
| SC3 | *Saccharomyces cerevisiae* | MN648817 | 100 |
| SC4 | *Saccharomyces cerevisiae* | MN648818 | 99 |
| SC5 | *Saccharomyces cerevisiae* | MN648819 | 100 |
| SC6 | *Saccharomyces cerevisiae* | MN648820 | 100 |
| SC7 | *Saccharomyces cerevisiae* | MN648821 | 100 |
| SC8 | *Saccharomyces cerevisiae* | MN648822 | 100 |
| SC9 | *Saccharomyces cerevisiae* | MN648823 | 100 |
| SC10 | *Saccharomyces cerevisiae* | MN648824 | 100 |
| SC11 | *Saccharomyces cerevisiae* | MN648825 | 100 |
| SC12 | *Saccharomyces cerevisiae* | MN648826 | 100 |
| SC13 | *Saccharomyces cerevisiae* | MN648827 | 100 |
| SC14 | *Saccharomyces cerevisiae* | MN648828 | 99 |
| SC15 | *Saccharomyces cerevisiae* | MN648829 | 100 |
| SC16 | *Saccharomyces cerevisiae* | MN648830 | 100 |
| SC17 | *Saccharomyces cerevisiae* | MN648831 | 100 |
| SC18 | *Saccharomyces cerevisiae* | MN648832 | 100 |
| SC19 | *Saccharomyces cerevisiae* | MN648833 | 100 |
| PK1 | *Pichia kudriavzevii* | MN648834 | 100 |


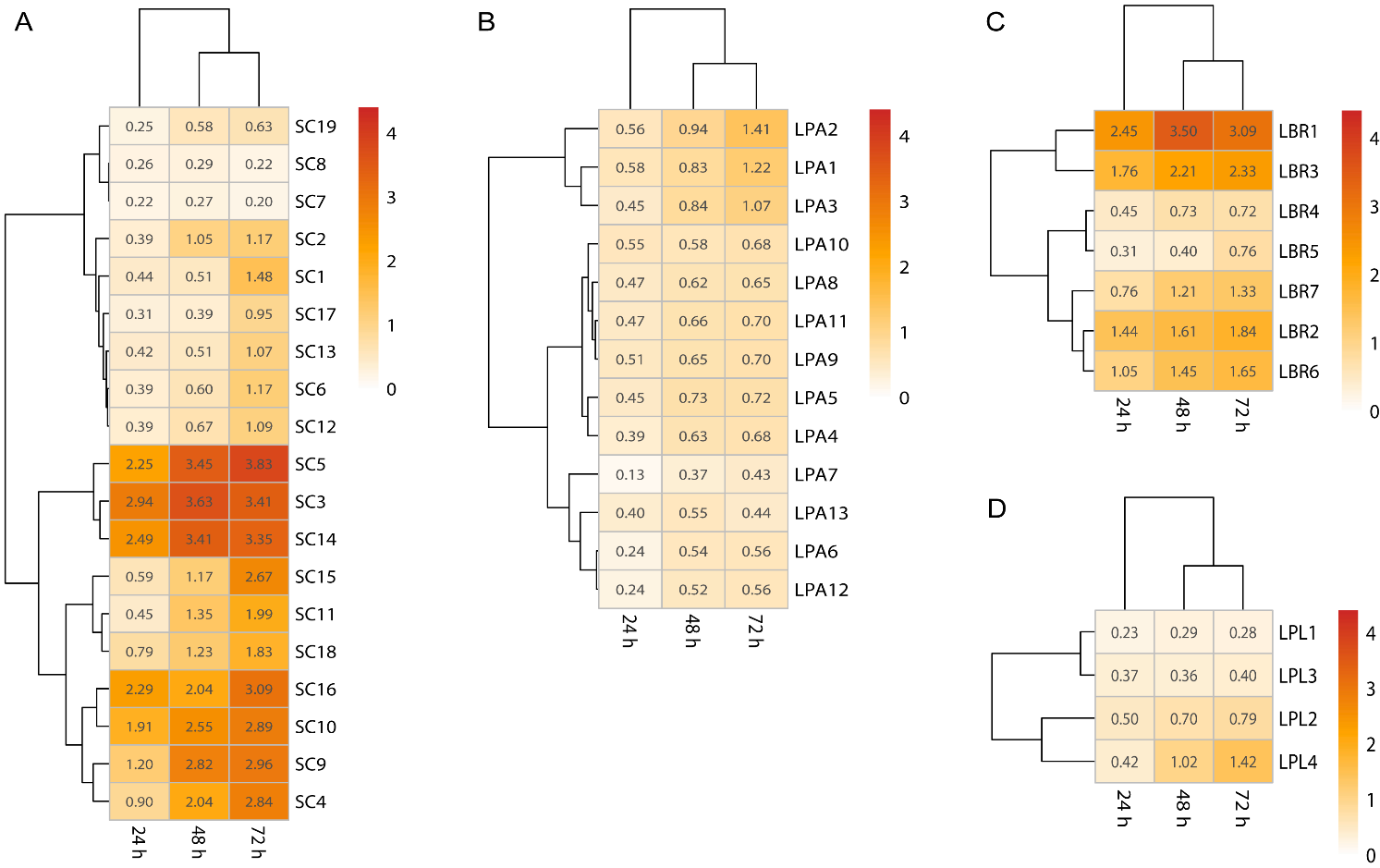


Figure S1. Single-species biofilms with *Daqu* media formed by *S. cerevisiae* (A), *L. paralimentarius* (B), *L. brevis* (C) and *L. plantarum* (D). LBR: *L. brevis*; LPA: *L. paralimentarius*; LPL: *L. plantarum*; SC: *S. cerevisiae*.


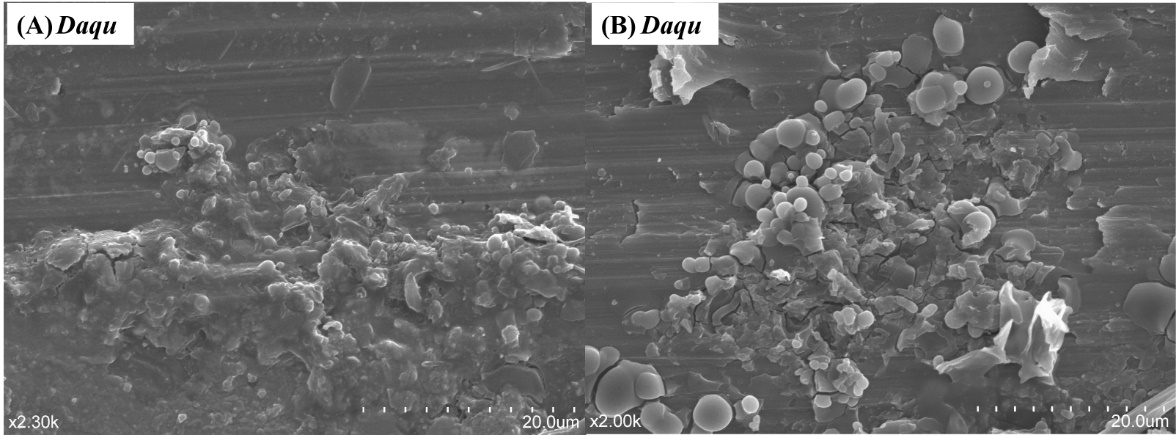


Figure S2. Scanning Electron Microscope (SEM) images of *Daqu* sample.
